# Grandparental Support and Maternal Postpartum Mental Health: A Review and Meta-Analysis

**DOI:** 10.1007/s12110-023-09440-8

**Published:** 2023-02-08

**Authors:** Madelon M.E. Riem, Marian J. Bakermans-Kranenburg, Maaike Cima, Marinus H. van IJzendoorn

**Affiliations:** 1 Behavioural Science Institute, Radboud University, Montessorilaan 3, 6525 HR Nijmegen, The Netherlands; 2 Clinical Child & Family Studies, Faculty of Behavioral and Movement Sciences, Vrije Universiteit, Amsterdam, The Netherlands; 3 Research Department of Clinical, Educational and Health Psychology, Faculty of Brain Sciences, UCL, University of London, London, UK

**Keywords:** Maternal postpartum mental health, Grandparents, Support, Childcare involvement

## Abstract

Support from grandparents plays a role in mothers’ perinatal mental health. However, previous research on maternal mental health has mainly focused on influences of partner support or general social support and neglected the roles of grandparents. In this narrative review and meta-analysis, the scientific evidence on the association between grandparental support and maternal perinatal mental health is reviewed. Searches in PubMed, EMBASE, MEDLINE, Scopus, and PsycINFO yielded 11 empirical studies on *N* = 3381 participants, reporting on 35 effect sizes. A multilevel approach to meta-analysis was applied to test the association between grandparental support and maternal mental health. The results showed a small, statistically significant association (*r* = .16; 95% CI: 0.09–0.25). A moderator test indicated that the association was stronger for studies reporting on support from the maternal grandmother in particular (*r* = .23; 95% CI: 0.06–0.29). Our findings suggest that involved grandparents, in particular mother’s own mother, constitute a protective factor for the development of maternal postpartum mental health problems. These findings have clear implications for interventions. Future studies should examine whether stimulating high-quality support from grandparents is a fruitful avenue for enhancing maternal postpartum mental health.

Perinatal mental health problems are common complications of pregnancy and the postpartum period and can have negative effects on both mother and child. In Western societies, research points to prevalence rates of 7–13% for depression during pregnancy and 10–15% in the early postpartum period (Bennett et al., 2004; Gavin et al., 2005). Other affective disorders, such as anxiety disorders (Fairbrother et al., 2016), also show high prevalence rates during the perinatal period (Howard et al., 2014), suggesting that pregnancy and the first months after birth are among the most vulnerable times in a woman’s life. Low social support has been identified as an important risk factor for developing postpartum mental health problems (Razurel & Kaiser, 2015), and in particular postpartum depression (Robertson et al., 2004). According to the adaptationist hypothesis for postpartum depression (PPD), PPD is viewed as a strategic adaptation to a lack of social support (Hagen, 1999). More specifically, mothers may develop PPD in response to a perceived lack of social support necessary to raise their child, resulting in low maternal investment in infants which in turn elicits greater levels of investment and support from others, such as fathers, grandparents or other family members. Although beneficial influences of partner support are well-documented (Yim et al., 2015), influences of grandparental support on postpartum maternal mental health received less attention, which is surprising given that grandparents, in particular the maternal grandmother, often play a role in newborn care (Aubel, 2021; Scelza & Hinde, 2019). In the current study, we therefore review available evidence on the association between grandparental support and maternal perinatal mental health and conduct a meta-analysis to test whether the association is replicable across studies.

Mothers typically do not rear children on their own, but share child care with other family members. From an evolutionary approach, family-shared childcare has even been suggested to be crucial for infant survival, because, according to calculations of evolutionary anthropologists, raising a child from birth to adulthood requires more than 13 million calories to be provided by caregivers (Kaplan, 1994), which is far more than a mother, or a father, can solely provide. This cooperative breeding aspect of human childrearing means that grandparents are likely to have contributed substantial amounts of care, time, and advice to their families throughout human history (Hrdy, 2009). Indeed, in a recent review, Aubel (2021) presented evidence that grandmothers play a coordinating role in newborn care in a large number of non-Western countries. For example, grandmothers advise mothers on breastfeeding or care of sick infants, often during an educational period of seclusion and isolation. In Western societies, grandmothers also influence maternal breastfeeding practices, for a review see (Negin et al., 2016). Grandparental involvement in childcare may lower the burden of childcare and may also support mothers’ return to work, thereby facilitating an adaptive transition to motherhood. Moreover, in a recent study, we found that support from grandparents was related to better maternal mental health and more adequate caregiving practices during the COVID-19 lockdown in China (Guo et al., 2021; Riem et al., 2021).

Support from grandparents may not only be beneficial for mothers but also for children. Ample empirical evidence has shown that grandparental involvement in childcare is positively associated with children’s socio-emotional and cognitive development (Sadruddin et al., 2019). These positive effects for children may be particularly important under adverse circumstances, such as parental illness. Indeed, a longitudinal study showed that a strong emotional connection to grandparents attenuates the risk of intergenerational transfer of maternal depression, indicating that grandparents have an important buffering effect (Silverstein & Ruiz, 2006). The pathway of grandparental influence on children can either be direct through positive grandparent-child interactions, or indirect through emotional and practical support of parents, which in turn enhances family well-being (Dunifon, 2013). For example, Spieker and Bensley (1994) showed that co-residence with grandmother indirectly impacted on children of teenage mothers. More specifically, teenage mothers who lived with their grandmother showed a higher quality of mothering and their infants were more likely to be securely attached compared to teenage mothers who did not benefit from the presence of a co-residential grandmother. Together, these studies indicate that involved grandparents can be an important source of support for both mothers and grandchildren.

However, support from grandmother is not a fail-safe remedy. When the relationship between mother and grandparents is conflictual or when grandparents interfere with parental childrearing, grandparents’ involvement may have negative effects on mothers’ affective state (Aubel, 2021; Coall & Hertwig, 2010). Conflicts with grandparents, most notably the paternal grandmother, have even been associated with increased risk for PPD (Lau & Wong, 2008) and can increase family stress which may negatively impact on mothers’ parenting practices (Dunifon, 2013). Indeed, one study found that African American adolescent mothers experience less parenting satisfaction when they had a confrontational relationship with their own mother (Hess et al., 2002). Furthermore, grandparent effects may depend on contextual factors, such as adverse or at-risk conditions. Beneficial effects may be more pronounced for mothers with higher needs of support, such as adolescent mothers who are at increased risk for developing PPD (Mollborn & Morningstar, 2009). Indeed, teenage mothers who receive high levels of support from grandmother show a higher quality of mothering and have higher chances of a secure mother-infant attachment relationships than teenage mothers with low grandmaternal support (Spieker & Bensley, 1994). However, it should be noted that under very adverse conditions, such as extreme poverty, presence of grandparents may be less beneficial and may even reduce life expectancy of offspring because they use scarce resources (Strassmann & Garrard, 2011).

In addition to contextual factors, the genetic relationship with grandparent may also matter in the effects of grandparental involvement. Influences of grandparents may depend on whether the support comes from maternal or paternal grandparents or from grandmother or grandfather. In particular, the maternal grandmother has been suggested to be an important source of support and advice (Aubel, 2021; Coall & Hertwig, 2010), which fits with evolutionary theories that emphasize the importance of the maternal grandmother for reproductive success. Grandmaternal support has been suggested to “lighten the reproductive load” of breeders, for example by helping with food provisioning during the post weaning phase. This enhances parental well-being and allows parents to reproduce earlier, more frequently and more successfully (Lahdenperä et al., 2004). A question that will be addressed in this meta-analysis is, therefore, whether in particular support from the maternal grandmothers is positively associated with maternal mental health.

In sum, support from grandparents, in particular the maternal grandmother, may play a role in mothers’ perinatal mental health. However, previous research on PPD mainly focused on influences of partner support or general social support and neglected the roles of caregivers within wider family systems. In the current study we therefore present a narrative review and a meta-analysis examining the association between grandparental support and maternal perinatal mental health. We were specifically interested in the maternal grandmother, since support from the own mother may be most beneficial (Aubel, 2021; Coall et al., 2018). In addition, we explore the moderating role of adverse context and differential effects of maternal grandmother versus (paternal) grandmother and grandfather. We expect that beneficial effects of grandparental involvement are more pronounced among mothers in high need for support, such as adolescent mothers, and that in particular support from the maternal grandmother predicts better maternal perinatal mental health.

## Methods

### Data Extraction

We systematically searched the databases PubMed, EMBASE, MEDLINE, Scopus, and PsycINFO. The search was finished in June 2021. The following search terms were used: (maternal OR mother*) AND (grandmaternal OR grandmother* OR grandfather* OR grandpaternal OR grandparent* OR grandparental) AND (perinatal* OR peri-natal* OR postnatal* OR post-natal* OR antenatal* OR ante-natal* OR postpartum* OR post-partum* OR peripartum* OR peri-partum* OR after birth) AND (stress* OR distress* OR depress* OR anxiety OR mental health). Studies were eligible if they examined and reported the association between grandparental presence, support, or involvement in childcare and maternal perinatal mental health symptoms, defined as the occurrence of all mental health problems (including depressive symptoms, anxiety, (parenting) stress/distress, general psychopathology) during pregnancy or the first postpartum year. Studies examining parenting stress were also included, because parenting stress is considered an indicator of mental health (Mikolajczak et al., 2018). Only empirical, peer-reviewed studies were included. Qualitative exploratory studies and theoretical reviews were excluded because they did not test associations with grandparental support. Dissertations and conference publications were also excluded because they were not peer-reviewed. Furthermore, studies examining maternal mental health later than one year postpartum were excluded. Titles and abstracts of all references resulting from the search were screened by two researchers. Full-texts were extracted for all references that were considered suitable or ambiguous. Review of full-texts of relevant studies was also performed by two researchers.

### Statistical Analyses

Effect sizes (Pearson’s *r*) were extracted for the studies that survived the screening procedure. Most studies reported more than one measure of grandparental involvement or reported mental health outcomes at various time-points. We therefore applied a multilevel approach and included a third level covering multiple outcomes within each study, in addition to the levels of participants and studies. The R packages meta, dmetar and metafor were used to analyze this three-level structure (Harrer et al., 2019). We performed moderator tests in order to examine potential differences in the effects of support of maternal grandmother versus (paternal) grandparents. We also tested the moderating role of the quality of the studies. A funnel plot was created and Egger’s test (Egger et al., 1997) was performed to visualize and test the potential presence of publication bias. In addition, the trim-and-fill approach (Duval & Tweedie, 2000) was used to compute a corrected effect size after imputing the presumably missing small studies with small effect sizes. Lastly, p-curve analysis was performed in order to detect potential bias due to *p*-hacking (Simonsohn et al., 2014).

## Results

**Fig. 1:**
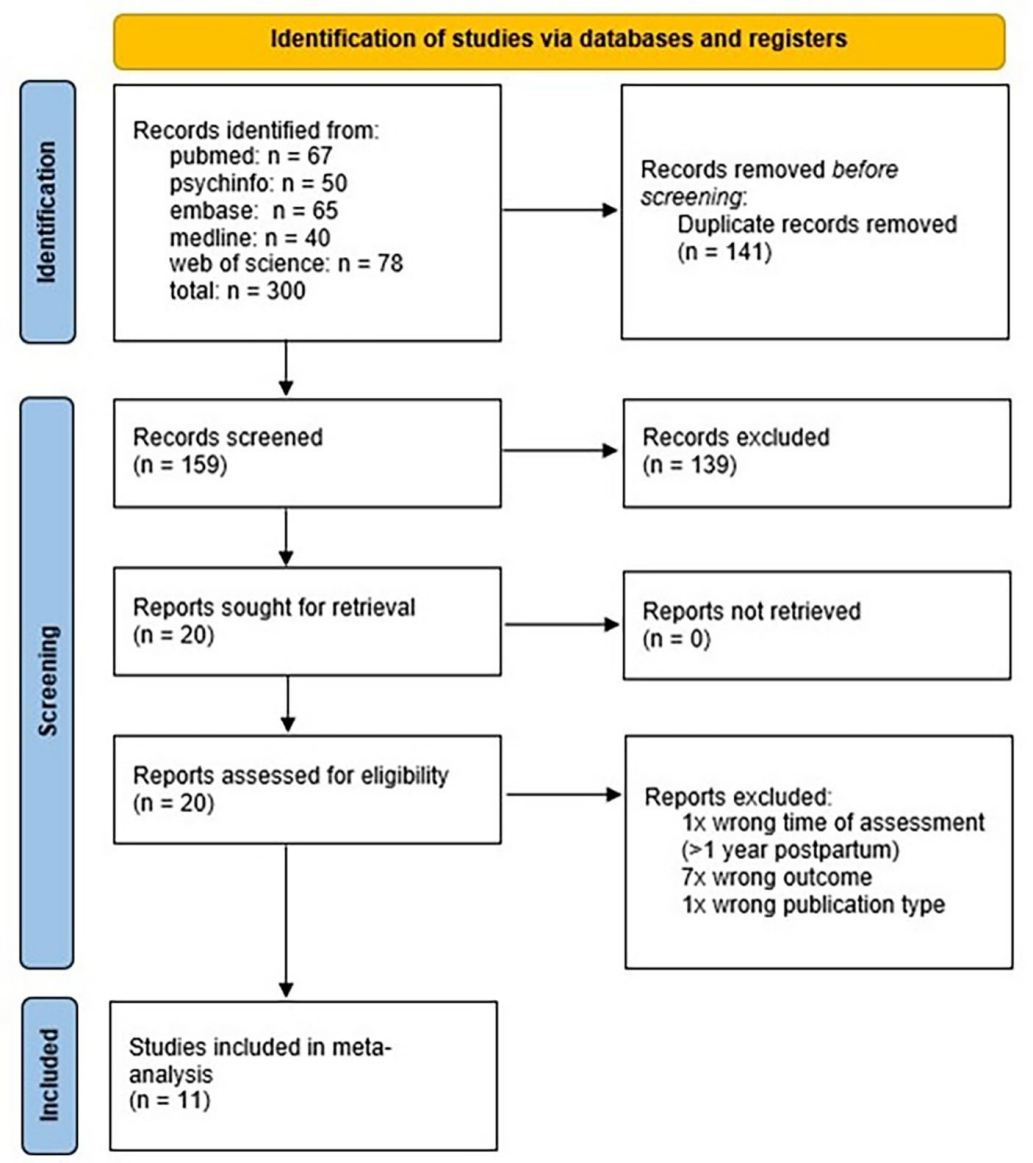
PRISMA flow diagram

*K* = 159 studies were identified after removal of duplicates. 139 studies were excluded based on title and abstract, and 20 studies were extracted and assessed for eligibility by full-text review, resulting in 11 studies meeting the inclusion criteria (Fig. 1). In Table 1, we present the characteristics of these studies, including risk of bias reflecting the quality of the study as based on five study characteristics (Shamseer et al., 2015). First, we checked whether inclusion and exclusion criteria for being in the study were prespecified and applied uniformly to all participants. Studies examining different levels of grandparental involvement (continuously versus dichotomously) also received a higher quality score. Third, we checked whether the variable measuring grandparental support or involvement was clearly defined, valid, reliable, and implemented consistently across all study participants. Similarly, studies with clearly defined, valid, and reliable mental health outcome measures received a higher quality score. Lastly, when grandparental support was measured prior to the measurement of the mental health outcome, studies received a higher score. Quality scores ranged from 2 to 5, with higher scores reflecting a higher quality.

**Table 1:** Characteristics of studies that survived the screening process

Reference	Country	*N*	Timing	Measurement of exposure: grandparent	Measurement of outcome: maternal mental health	Risk of bias
Arnold et al., 2011	USA	784 adolescent mothers	second trimester, third trimester, 6 months postpartum	Caregiving structure with or without involved grandparents assessed 6 months postpartum. No differentiation maternal and paternal grandparents.	12-item subscales of the short form of the Parenting Stress Index (PSI), assessed 6 months postpartum	3
Borcherding et al., 2005	USA	72 adolescent mothers	3 months postpartum	Grandparent responsiveness assessed with Grandparent Support Scale for Teenage Mothers. 86% maternal grandmother	Center for Epidemiologic Studies–Depression Scale (CES-D)	4
Findler et al., 2007^a^	Israel	148 mothers of twins (70 pre-term, 78 full-term)	after birth, 1 year postpartum	Support from maternal grandmother assessed with the Support Functions Scale, measured after birth	Mental Health Inventory, assessed 1 year postpartum	5
Noy et al., 2015^a^	Israel	414 mothers (overrepresentation preterm infants and twins)	1 year postpartum	Maternal grandmother’s emotional and instrumental support measured with adapted version of the Support Functions Scale	Mental Health Inventory	4
Porat-Zyman et al., 2018^a^	Israel	561 mothers (overrepresentation preterm infants and twins)	1 month, 1, 2, and 4 years postpartum	Maternal grandmaternal support measured with the Support Functions Scale, 1 month postpartum	Mental Health Inventory at 1, 2, and 4 years postpartum	4
Kalil et al., 1998	USA	194 adolescent mothers	6, 18, 30 months postpartum	Co-residence maternal grandmother assessed 6 months postpartum	Center for Epidemiologic Studies–Depression Scale (CES-D), assessed 6, 18, and 30 months postpartum	3
Leadbeater & Linares 1992^b^	USA	120 adolescent mothers	1, 6, 12, and 28–36 months postpartum	Acceptance by maternal grandmother assessed 1 month postpartum with the maternal subscale of the Mother-Father-Peer Scales	Beck Depression Inventory (BDI), assessed 1, 6, and 12 months, and 28–36 months postpartum	4
Way & Leadbeater 1999^b^	USA	120 adolescent mothers, age 13–19 years	1, 6, and 12 months, and 6 years postpartum	Number of months lived with maternal grandmother during first postpartum year, child care assistance from grandmother measured at 12 months	The Beck Depression Inventory (BDI), averaged for 1-, 6-, and 12-month assessment	2
Lee et al., 2020	USA	192 young African American mothers	4 months postpartum	General social support from primary parent figure (PPF) assessed with a 6-item questionnaire and direct infant care support from PPF assessed with an 8-item questionnaire. 74% maternal grandmother	Center for Epidemiologic Studies–Depression Scale (CES-D), “difficult child” subscale of the Parenting Stress Inventory Short Form (PSI-SF)	4
Leung & Lam 2012	Hong Kong	156 mothers: 78 assigned to co-parenting intervention, 78 assigned to control intervention	T1: 14–28 weeks gestation and before intervention, T2: pregnancy and after intervention, T3: 6–8 weeks postpartum	Group antenatal intervention targeting intergenerational conflicts with grandparents. No differentiation maternal and paternal grandparents.	4-item perceived stress scale and the 10-item Edinburgh Postnatal Depression Scale (EPDS), assessed at T1, T2, T3	3
Wang et al., 2017	China	1126 mothers	14–60 days postpartum	Living situation: (a) Only with husband, (b) With parents, (c) With parents-in-law. No differentiation with maternal grandmother.	Edinburgh Postnatal Depression Scale (EPDS)	3

^a,b^ Studies with partly overlapping samples

The 11 studies that survived the screening process are discussed below, with the specific measures of grandparental involvement. We found that four studies examined the association between co-residence with grandparents and maternal perinatal mental health. A total of seven studies measured grandparental support continuously in order to examine the role of quantity and quality of support from grandparents.

### Studies Examining Caregiving Roles and Co-Residence of Grandparents

In a study of *N* = 784 adolescent mothers in the USA, Arnold and colleagues (2011) examined the association between grandparental childcare involvement and maternal postpartum stress levels as assessed with the Parenting Stress Index (PSI) (Abidin, 1997) at six months postpartum. Mothers reported whether she, the father, and/or the grandparents were caregivers. Results showed that maternal parenting stress was lowest among mothers who shared childcare with father or with father and grandparents and higher among mothers who were the only caregiver or who shared childcare with grandparents but not with father. This indicates that sharing childcare with multiple family members promotes maternal mental health. No differences in maternal parenting stress were found between mothers who received support from grandparents compared to mothers who did not receive support from grandparents. This study, however, did not differentiate between paternal and maternal grandparents or between grandmother and grandfather. Another limitation was that this study did not measure the level and the quality of grandparental involvement.

Another study in the USA examined the association between co-residence with grandmother and maternal depression in a group that can be considered at risk for postpartum depression, that is, adolescent mothers. Kalil and colleagues (1998) measured maternal depressive symptoms, assessed with the CES-D (Radloff, 1977), in a sample of *n* = 194 adolescent mothers in the USA. The authors found that co-residence with maternal grandmother was related to lower levels of maternal depressive symptoms at six months postpartum. Follow-up analyses revealed a significant interaction between co-residence and family cohesion (i.e., degree of closeness among family members and feeling respected by family), indicating that mothers who lived with grandmother only experienced fewer depressive symptoms when there was positive family cohesion. A limitation of this study was that the association between grandmaternal co-residence and depressive symptoms was measured cross-sectionally. In addition, the quality and the level of grandmaternal involvement were not assessed.

In a sample of 120 adolescent mothers, Way and Leadbeater (1999) examined whether the number of months living with maternal grandmother during the first postpartum year and childcare assistance from grandmother assessed at one year postpartum were related to maternal depressive symptoms averaged across three time points across the first postpartum year (one month, three months, and one year postpartum). Depressive symptoms were measured with the BDI (Beck et al., 1988). The number of months lived with grandmother was not significantly related to depressive symptoms. The negative correlation between childcare assistance from grandmother and depressive symptoms was also not significant (*p* < .10). A limitation of the study was that the time of living with grandmother was not measured continuously, but was collapsed into three scores representing 1 or 2 months, 3 to 9 months, and 10 to 12 months. Moreover, direct support in childcare was measured dichotomously (regular childcare assistance versus occasional or no childcare assistance).

In a cross-sectional study in China, Wang et al. (2017) examined whether living with parents or living with parents-in-law was related to risk for postpartum depression. A total of 1152 mothers reported on their living situation (living with husband, living with parents, or living with parents-in-law) 14–60 days postpartum. Depressive symptoms were assessed with the Edinburgh Postnatal Depression Scale (EPDS) (Cox et al., 1987). The study showed that mothers who lived with their parents-in-law were at higher risk of PPD than mothers who lived with their husband only. Mothers living with their parents did not significantly differ in PPD symptoms compared to mothers living with their husband only. The study did not differentiate between living with grandmother or grandfather. Level and the quality of grandparental involvement in childcare were not assessed.

To summarize, results of studies examining the association between grandparental presence and maternal mental health are somewhat mixed. Childcare assistance from grandparents seems to promote maternal mental health, although the genetic relationship with grandparent matters. Whereas co-residence with the maternal grandmother has beneficial effects, the presence of paternal grandparents in the household can also increase risk for maternal mental health problems.

### Studies Examining Quantity and Quality of Grandparental Support

Borcherding and co-authors (2005) examined the association between grandparent support as assessed with the Grandparent Support Scale for Teenage Mothers (GSSTM) and maternal depressive symptoms assessed with the Center for Epidemiologic Studies–Depression Scale (CES-D) (Radloff, 1977) at three months postpartum. The sample consisted of *N* = 72 adolescent mothers from the USA. The purpose of the study was to describe the initial psychometric testing of the teen version of the GSSTM, an instrument that focuses on adolescent mothers’ perception of parental support. The authors found a significant negative association between grandparental support and depressive symptoms, indicating that mothers who received high quality support experienced fewer depressive symptoms. This is in line with our hypothesis that grandparental involvement promotes maternal mental health. However, grandparent’s decision-making regarding childcare and interfering behaviors were related to increased depressive symptoms. Although the study did not differentiate between maternal and paternal grandparents, 86% of mothers reported on support from the maternal grandparent.

In a longitudinal study, Findler and colleagues (2007) examined the contribution of grandmother’s support following delivery to the psychological mental health of first-time and non-first-time Israeli mothers of preterm (*n* = 70) and full-term (*n* = 78) twins, 1 year postpartum. Support from maternal grandmother was assessed with the Support Functions Scale (Dunst et al., 1988) after childbirth and maternal affective state was measured with the Mental Health Inventory (MHI) (Veit & Ware, 1983) one year postpartum. The study showed that support from grandmother was significantly related to better maternal mental health among mothers with preterm twins, but not among mothers with full-term twins. This is consistent with our hypothesis that grandparental support is particularly important for mothers in high need of support.

In a partly overlapping sample of Israeli mothers, Noy and colleagues (2015) also examined the association between grandmaternal support and maternal postpartum mental health. The sample was overrepresented with mothers of twins or (singleton) preterm infants, but also included full-term singletons. A total of 414 mothers reported on emotional and instrumental support from their own mother, measured with an adapted version of the Support Functions Scale one year postpartum. Maternal mental health was assessed with the MHI, one year postpartum. Consistent with our hypothesis, the authors found that grandmother’s emotional support and instrumental support were both significantly related to better maternal mental health. A limitation of the study was the cross-sectional design.

In another study in Israel, partly overlapping with the study by Noy et al. (2015) and Findler et al. (2007), Porat-Zyman and colleagues (2018) examined whether support from grandmother assessed with the Support Functions Scale was related to maternal mental health assessed with the MHI. A longitudinal design was used to examine the contributions of internal resources, such as adult attachment style, and external resources, including maternal grandmother’s support, to maternal mental health over the course of 4 years following childbirth. The sample consisted of 561 mothers with an overrepresentation of mothers of preterm infants and twins. Support from grandmother was assessed with the Support Functions Scale one month postpartum, and maternal mental health was assessed with the MHI one month, one year, two years, and four years postpartum. The authors employed a latent growth curve model within structural equation modeling to examine the factors associated with postpartum maternal mental health at one month postpartum and changes during the first four years postpartum. They found that support from grandmother was not significantly related to maternal mental health at one month postpartum or improvement across the first postpartum years, which seems to contrast with the studies by Noy et al. (2015) and Findler et al. (2007). One explanation for this discrepancy is that the study focused on changes in maternal mental health, at multiple time points extending beyond the first year postpartum. This study reported the same outcome measure of maternal mental health, at the same period of assessment, with almost the same sample as Noy et al. (2015), but no meta-analytically useable outcome data were reported. We therefore only included the study of Noy et al. (2015) in our meta-analysis.

Leadbeater and Linares (1992) examined how acceptance by grandmother assessed at one month postpartum was related to adolescent mothers’ depressive symptoms at one, six and 12 months postpartum. The sample partly overlapped with the sample of the study conducted by Way and Leadbeater (1999). 120 adolescent mothers from the USA completed the maternal subscale of the Mother-Father-Peer Scales (Epstein, 1983) to report on grandmaternal acceptance, reflecting emotional support, at one month postpartum and completed the Beck Depression Inventory (BDI) (Beck et al., 1987) at 1, 6, and 12 months postpartum. The study showed that grandmaternal acceptance was negatively related to mothers’ depressive symptoms at the three time points, which is in line with our hypothesis. A limitation of this study was that mothers reported on the quality of the relationship with their mother during their childhood. However, the authors reason that, although the Mother-Father-Peer Scales is a retrospective assessment, given their young age (13–19 years) and continued co-residence with own mother, the adolescent mothers’ responses likely also reflect current experiences.

In a study with 192 adolescent African American mothers, Lee et al. (2020) examined the associations between social support and depressive symptoms and parenting stress at four months postpartum. General social support from the primary parent figure, typically the grandparents, was assessed with a self-developed 6-item questionnaire and direct infant care support from grandparents was assessed with a self-developed 8-item questionnaire. Maternal depressive symptoms were assessed with the CES-D and the Difficult Child subscale of the Parenting Stress Inventory Short Form (PSI-SF) (Abidin, 1997) was used to measure parenting stress. The authors report a significant negative correlation between general support from grandparents and parenting stress, indicating that support from grandparents is related to lower stress levels in these mothers. Direct support with childcare was, however, not significantly related to stress levels. Neither was there a significant correlation between general support and maternal depressive symptoms. The correlation between direct support with childcare and maternal depressive symptoms was not significant. The study did not differentiate between different categories of grandparents (maternal or paternal), although the majority of mothers (74%) reported on support received from their own mother.

Only one study examined the effects of grandparental involvement on maternal mental health with a randomized controlled trial (RCT). In an RCT, Leung & Lam (2012) examined the efficacy of an antenatal group intervention targeting intergenerational conflicts with grandparents. The intervention consisted of four weekly group sessions of interpersonal psychotherapy aiming at reducing stress and depressive symptoms in new mothers and enhancing happiness and self-efficacy in managing intergenerational conflict in childcare. The sample consisted of 156 mothers from Hong Kong who were assigned to the intervention group (*n* = 78) or a control group (*n* = 78) that received care as usual. Maternal stress was measured with the Perceived Stress Scale (Cohen, 1986) and depressive symptoms were assessed with the EPDS before the intervention at 4- to 28-weeks gestation (T1), after the intervention later in pregnancy (T2), and at 6- to 8-weeks postpartum (T3). Mothers who received the intervention showed significantly reduced stress levels at T2 and T3 compared to T1, while the control group reported increased stress levels over time. At T2, stress levels of mothers in the intervention group were significantly lower compared to the control group. A similar pattern was observed for depressive symptoms. The intervention group showed a reduction of depressive symptoms over time, whereas the control group reported increased depressive symptoms at T2 and T3 compared to T1. The control group showed a greater reduction in happiness from baseline to T2 and T3 compared with the intervention group, with a significant group difference at T2. Perceived health ratings changed minimally over time and there were no significant group differences. The findings of this study indicate that resolving conflicts about childcare with grandparents reduces stress and depressive symptoms in mothers, which is in line with our hypothesis that high-quality grandparental involvement promotes maternal mental health. The use of an RCT is a strong methodological argument for a causal role of grandparents in promoting maternal mental health. However, a limitation of the study was that the authors did not differentiate between paternal and maternal grandparents or between grandmother and grandfather. In addition, health was measured with a single-item questionnaire measuring perceived health.

To summarize, studies examining quantity and quality of grandparental support point to beneficial effects of emotional and practical grandparental support for mothers’ mental health. These beneficial effects of grandparental support are not only observed in adolescent mothers who are at risk for depression and are in high need of support, but also in mothers in low risk conditions. However, quality of support seems to matter in the association between grandparental support and maternal health. Whereas high-quality support promotes maternal mental health, intrusive involvement of grandparents may increase risk for maternal mental health problems.

### Meta-analysis

**Fig. 2:**
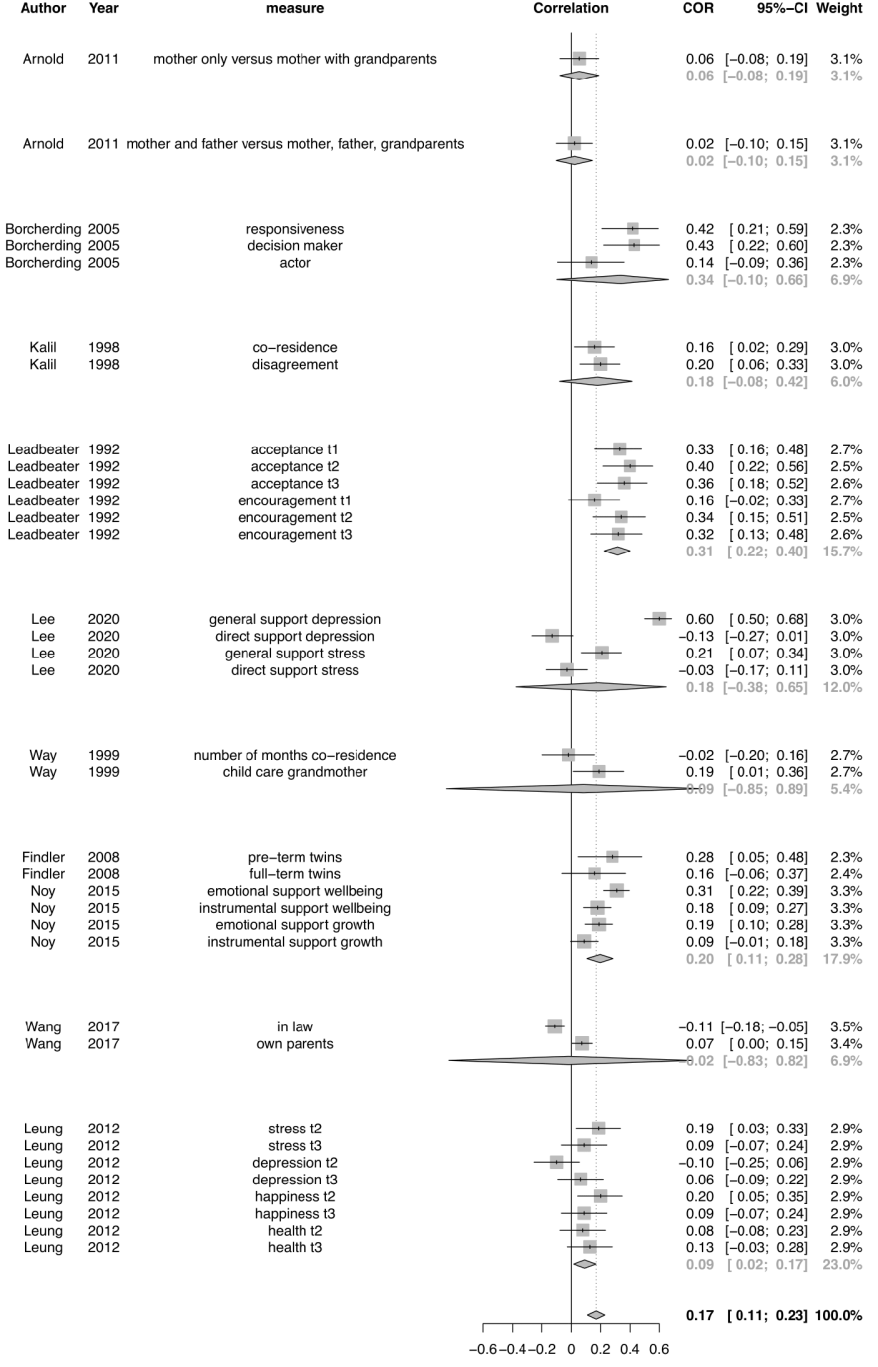
Forest plot of the 35 effect sizes included in the meta-analysis

The meta-analysis aimed to test the association between grandparental support and maternal perinatal mental health. The analyses were based on 10 eligible studies, including 3381 participants and reporting on 35 effect sizes (see Fig. 2 for a forest plot). The multilevel meta-analysis estimated the overall effect size of a full model with three levels to be *r* = .16 (*SE* = 0.04; 95% CI: 0.09–0.25; *t*_34_ = 4.28, *p* < .001). After removing the study level, the LRT test was not significant (*p* = .225). Although this indicates that a two-level model would also fit the data, we kept the three-level structure, since it has been suggested that this is a more adequate representation of how the data are generated (Harrer et al., 2019). Moderator tests showed significant differences in studies reporting the effects of support of maternal grandmother versus (paternal) grandparents. The association between grandparental support and mental health was stronger for studies reporting on support from specifically the maternal grandmother (*r* = .23, *SE* = 0.06; 95% CI: 0.06–0.29, *F*_1,33_ = 9.35, *p* = .004). The quality of studies was also a significant moderator, with stronger associations between grandparental involvement and mental health when the quality of study was lower (*F*_1,33_ = 7.41, *p* = .010). We also tested whether effect sizes were stronger for adolescent mothers compared to adult mothers, as adolescent mothers are in high need for support. However, age of mother was not a significant moderator (*F*_1,33_ = 1.79, *p =* .19).

**Fig. 3:**
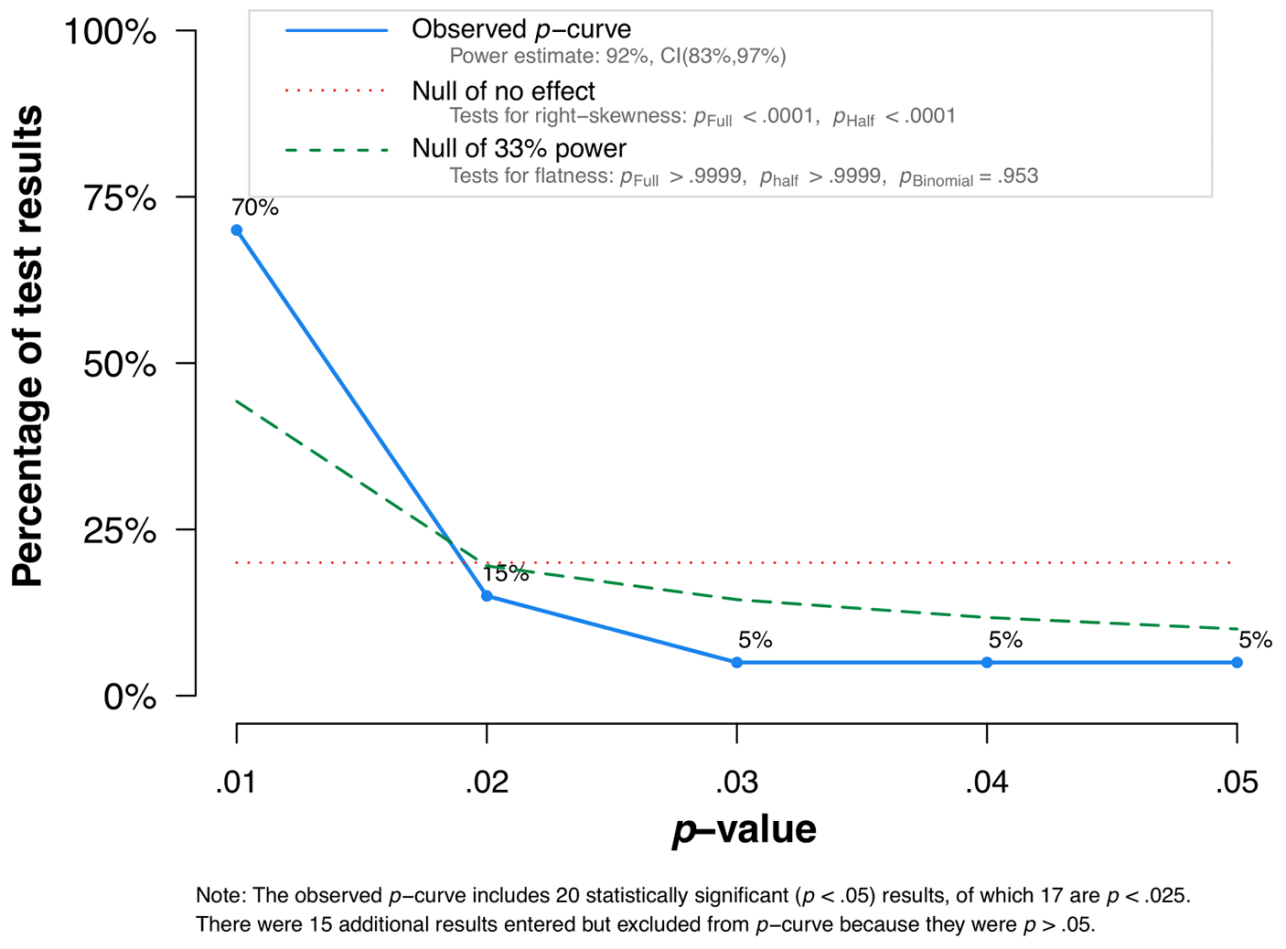
The *p*-curve analysis performed on *k* = 20 significant effect sizes

A funnel plot revealed a publication bias and Egger’s test showed that this bias was significant (intercept = 3.17, *p* = .008). The trim-and-fill approach was applied in order to compute a corrected effect size after imputing the 12 missing small studies with small effect sizes. After trim-and-fill, the overall effect size was estimated at *r* = .07 and was not significant (*p* = .052). P-curve analysis was performed on *k* = 20 significant effect sizes in order to examine potential bias due to p-hacking. The total number of effect sizes with *p* < .025 was *k* = 17 (48.57%). The test of right-skewness was significant, while the flatness test was not significant (see Fig. 3), indicating that the data contain evidential value. As a robustness test, the trim-and-fill approach was repeated with studies reporting on support from specifically the maternal grandmother (7 studies, 23 effect sizes). Results showed a significant overall effect size of *r* = .17 (*p* < .001) after the imputation of 5 missing small studies with small effect sizes.

## Discussion

We examined the association between grandparental support and maternal perinatal mental health in a narrative review and a meta-analytic study, with a particular focus on the role of the maternal grandmother in promoting maternal mental health. Our meta-analysis showed that grandparental support is related to better maternal mental health in the first year postpartum. In line with our expectations, we found that, in particular, support from the maternal grandmother was associated with better maternal mental health. A stronger overall effect size was found for studies reporting on support from the maternal grandmother, indicating that support from mother’s own mother is most beneficial. Importantly, the association between grandparental involvement and maternal mental health was not moderated by adverse conditions. Beneficial effects of grandparental support were not only observed in adolescent mothers who are at risk for depression and are in high need of support, but also in mothers in low risk conditions. Our narrative review suggests that both practical and emotional support from grandparents has beneficial effects, although the quality of the relationship with grandparents may matter in the association between grandparental support and maternal mental health. Our findings indicate that involved grandparents are an important source of practical and emotional support for mothers and that presence of involved grandparents constitutes a protective factor for the development of maternal postpartum mental health problems.

Our finding that specifically support from the maternal grandmother contributes to postpartum maternal mental health fits with evolutionary theories that explain the importance of the maternal grandmother for reproductive success. Research has shown that grandmaternal care is related to reproductive success and number of children in pre-industrial societies (Engelhardt et al., 2019), a finding that has been interpreted as support for the Grandmother Hypothesis, which states that the prolonged post-reproductive lifespan of grandmothers is the outcome of adaptive benefits gained by assisting their own offspring to reproduce successfully (Hawkes, 2004; Hawkes et al., 1998). Grandmothers who live long enough to offer a helping hand with childcare allowed their daughters to have more children, resulting in the transmission of longevity genes to the next generation. Our finding that in particular grandmaternal support contributes to better postpartum maternal mental health indicates that involvement of grandmother in postpartum care still has benefits in contemporary societies. Future studies should examine why the maternal grandmother has strongest beneficial effects. From an evolutionary perspective, it makes sense that mother’s own mother, rather than grandfathers and the paternal grandmother, offers postpartum support, given that the maternal grandmother is more certain of her genetic relationship to her grandchild. However, mothers may also benefit most from support from their own mother for other reasons than genetic relatedness, for example because they feel closer to their own mother and feel more comfortable in the presence of their own mother.

High-quality involvement of grandparents may not only benefit mother’s mental health, but also the child. The adverse effects of postpartum depression on maternal-infant interaction have been well-documented and indicate that mothers with PPD are less emotionally available or affectively unresponsive and that mother-child interactions are less synchronous (Beck, 1995; Field et al., 1990). These problematic interactions can in turn negatively affect children’s cognitive and emotional development (Beck, 1998; Grace et al., 2003). Social support is generally associated with improved mother-infant bonding and can buffer the negative effects of poor maternal mental health on the mother-infant relationship (McNamara et al., 2019). Studies examining the effects of interventions targeting social support also report positive effects of support from peers or partner on mothers’ mental health and childcare abilities (Dennis, 2003; Fleming et al., 1992; Kettrey & Steinka-Fry, 2021; Misri et al., 2000), but neglect the role of grandparents as a source of support. In contrast to peers or social support groups that provide mostly emotional support to new mothers, grandparents can also contribute with practical support through direct involvement in childcare, thereby lowering stress and caregiving load for mothers. Future studies should therefore examine the impact of grandparental emotional and practical support in the treatment of mothers suffering postpartum mental health problems. Involving grandparents in treatment may add to the effectiveness of existing intervention programs.

Although high-quality support from grandparents promotes maternal mental health, intrusive involvement of grandparents may negatively affect maternal mental health. Moreover, our narrative review suggests that, for some mothers, co-residence with paternal grandmothers increases risk for depressive symptoms, possibly because of grandparental intrusiveness or a conflictual relationship. Hence, the effects of grandparental support are likely to be a function of the quality of grandparental involvement in childcare and the way childcare is shared between mother and grandparents. Interventions should therefore not only devote attention to possibilities for enhancing quantity of grandparental involvement, but also quality of co-parenting with grandparents and the intergenerational relationship. To our knowledge, only one study examined the effectiveness of an intergenerational co-parenting intervention in reducing maternal mental health problems with a randomized controlled trial. Leung & Lam (2012) showed that a short program aiming at enhancing self-efficacy in managing intergenerational conflict in childcare was effective in reducing stress and enhancing happiness among new mothers in Hong Kong specifically when mothers experienced high levels of depressive symptoms. High-quality co-parenting with grandparents may be particularly important in the Chinese culture, where parents share childcare responsibilities with highly involved co-residential grandparents (Luo et al., 2020). However, it should be noted that the majority of studies included in our meta-analysis was conducted in the USA, possibly indicating that grandparents also exert protective effects on maternal mental health in Western societies. Western maternal mental health policies may therefore also benefit from involving grandparents in treatment programs, but generally ignore the role of grandparents and primarily focus on strengthening maternal and infant care services.

The overrepresentation of USA samples in the present meta-analysis can be considered a limitation. High-quality support from grandparents may be particularly important for mothers in countries where formal childcare is not readily available and co-residence with grandparents is common. Future studies should therefore examine the role of grandparents in non-Western societies. Future studies should also examine the role of grandparental support in promoting maternal mental health during pregnancy, as the majority of previous studies focused only on the postpartum period. It should, however, be noted that the use of additional search terms such as pregnancy" and “prenatal” might have resulted in more studies focusing on the prenatal period without extension to the postnatal period. Furthermore, it should be noted that our meta-analysis included only 10 studies, which is a limitation of the current study. Due to the limited number of studies, it is possible that our meta-analysis was underpowered to detect moderator effects such as adverse condition. Moreover, the effect size was small and there was publication bias. After the trim-and-fill procedure for publication bias, the overall effect size of grandparental support was not significant (*p* = .052).

Another evident limitation of the study is that no causal conclusions can be drawn. It is tempting to think that a lack of support from grandparents contributes to the development of depressive symptoms in mothers, but poor maternal mental health may also interfere with mothers’ ability to mobilize support from grandparents and/or benefit from it. Hence, the association between grandparental support and maternal postpartum mental health could be bidirectional. For example, research has shown that maternal depression limits mothers’ ability to engage in productive resolution of caregiving disagreements (Belsky et al., 1998; Feinberg, 2003), and hinders the ability to mobilize co-parental support with childcare (Negron et al., 2013). In fact, postpartum depression has been described as a call for social support to raise the child (Hagen, 1999), related to difficulties in reaching out in a more effective way. Future studies are needed to examine the direction of the association between grandparental support and maternal mental health, for example with longitudinal designs or experiments manipulating quantity and quality of grandparental involvement. Finally, based on the studies included in our meta-analysis, it was not possible to differentiate between the effects of support from grandmothers and grandfathers. Whereas several studies point to beneficial effects of support from grandmother, benefits of grandfathering are less clear (Coall et al., 2018). More research on the benefits of grandfathering is crucially needed to understand grandpaternal caregiving roles, as the study of grandfathering is still in its infancy.

To conclude, we found meta-analytic evidence for an association between grandparental support and better maternal mental health during the first year postpartum. Our findings call for more research on grandparental support, with particular attention to potential factors that may moderate grandparenting effects, such grandfather versus grandmother, culture, and contextual factors including number of children. Support from mother’s own mother was most beneficial, although the quality of the intergenerational relationship matters in the effects of grandparental support. While causality still needs to be tested in future research, the meta-analytic result that grandparental support is related to less maternal mental health problems has clear implications for interventions. Maternal mental health policies narrowly focus on the parents and the child and often ignore the role of caregivers in wider family systems. Future studies should therefore examine whether stimulating high-quality support from grandmother could be a fruitful/promising avenue for enhancing maternal mental health.
